# Forensic STR Loci and Schizophrenia: An Exploration of Implications for Forensic Applications and Genetic Privacy

**DOI:** 10.3390/genes15121525

**Published:** 2024-11-27

**Authors:** Qi Yang, Chun Yang, Zhiqi Hua, Qi Shen, Anqi Chen, Huajie Ba, Suhua Zhang

**Affiliations:** 1 Institute of Forensic Science, Fudan University, Shanghai 200032, China; myjasmine77@163.com (Q.Y.); zqhua24@m.fudan.edu.cn (Z.H.); qi_shen_2024@163.com (Q.S.); anqi_chen@fudan.edu.cn (A.C.); 2 The 904th Hospital (Changzhou Branch) of Joint Logistic Support Force of Chinese People’s Liberation Army, Changzhou 213000, China; yangchun8749@163.com; 3 School of Forensic Medicine, Shanxi Medical University, Jinzhong 030600, China; 4 DNA Laboratory, Public Security Bureau of Changzhou, Changzhou 213000, China

**Keywords:** short tandem repeat (STR), schizophrenia, association study, forensic DNA database, forensic ethics

## Abstract

Background/Objectives: Short tandem repeat (STR) loci are widely used in forensic genetics for identification and kinship analysis. Traditionally, these loci were selected to avoid medical associations, but recent studies suggest that loci such as TH01 and D16S539 may be linked to psychiatric conditions like schizophrenia. This study explores these potential associations and considers the privacy implications related to disease susceptibility. Methods: We analyzed 19 STR loci, including CODIS core loci and additional loci like Penta D and Penta E. Statistical analyses were conducted on a dataset of schizophrenia patients and matched control individuals to assess the relationship between STR polymorphisms and schizophrenia risk. Results: No significant associations were found between the 19 analyzed loci and schizophrenia in this dataset. While initial analyses revealed minor allele frequency differences at the D3S1358, D13S317, and TPOX loci between the schizophrenia and control groups, these differences did not retain statistical significance following Bonferroni correction (corrected *p* < 0.0026 for all loci). Conclusions: Although no significant associations were found between STR loci and schizophrenia, this study highlights the importance of considering the potential for forensic DNA data to reveal health-related information. As forensic DNA databases continue to expand, there is a growing need to reassess ethical and legal guidelines to ensure the protection of individual privacy. Future research should continue exploring these genetic associations with larger, more diverse samples to further understand their implications.

## 1. Introduction

Short tandem repeats (STRs) have been extensively used in forensic genetics, primarily for human identification (HID) and paternity testing. In 1997, the Federal Bureau of Investigation (FBI) introduced 13 core autosomal STR loci into the Combined DNA Index System (CODIS), which has since become a standard tool in forensic investigations worldwide [1]. With advances in DNA identification technology and the diversification of forensic needs, the number of CODIS STR loci was expanded to 20 in 2017 [2]. Beyond the core loci, commercial forensic kits, such as those including Penta D and Penta E, have further enhanced the discriminatory power of STR analysis, providing greater precision in a forensic context [1].

The selection of forensic STR loci was initially guided by the principle that these markers should not be associated with phenotypic traits or medical conditions [2]. This ensured that their use in forensic settings would focus solely on identification purposes, without infringing on personal medical privacy [3]. However, advances in genomic research have begun to challenge this foundational assumption. Non-coding regions of the genome, where many forensic STRs are located, have been increasingly recognized as playing critical roles in regulating gene expression and 3D chromatin architecture [4,5,6]. Variations within these non-coding regions, including STRs, may influence gene expression and contribute to phenotypic variation, raising concerns that forensic STR data might inadvertently reveal sensitive health information [7].

A 2020 review reported that 20 autosomal STRs among 24 forensic loci (including the CODIS core loci and additional markers) were associated with 50 published trait–disease associations [8]. Notably, schizophrenia emerged as the most frequently described trait, potentially linked to 14 loci (previous reviews identified 8 loci), including TH01 [9,10,11,12], D2S441 [13], FGA [14], vWA [15,16,17], D2S1338, D16S539, D18S51 [9], D8S1179 [17], D7S820, D19S433 [12], Penta D [18], D13S317, D5S818 [19], and D3S1358 [20]. These findings indicate that forensic STRs, traditionally used solely for identification, could potentially disclose genetic predispositions to health conditions.

Schizophrenia is a complex psychiatric disorder characterized by delusions, hallucinations, and cognitive impairments, affecting approximately 1% of the global population [21]. In forensic contexts, schizophrenia is particularly relevant, due to its association with aggressive behavior, violence, and higher rates of recidivism among offenders [22]. Given its clinical and forensic significance, understanding its genetic underpinnings is crucial for improving risk assessments and preventive strategies in forensic psychiatry [23].

Among the STRs associated with schizophrenia, TH01 has been the most frequently reported. TH01 is a tetrameric STR locus located in the first intron of the tyrosine hydroxylase (*TH*) gene. Studies have reported that allelic variations of TH01 may exert a quantitative silencing effect on *TH* gene expression [24]. The *TH* gene encodes tyrosine hydroxylase, the rate-limiting enzyme in dopamine synthesis, which is subsequently converted into norepinephrine and epinephrine [24,25]. These neurotransmitters are crucial for brain function and mood regulation. Beyond schizophrenia, TH01 has also been linked to other psychiatric conditions, such as depression [26,27], delusional disorder [28], suicidal behavior [29], and aggressive behavior [30,31,32,33].

Although these studies report statistically significant associations (*p* < 0.05), they generally do not explore underlying biological mechanisms or confirm the associations as independent causative or predictive factors [8]. Potential associations between STRs like TH01 and disease may result from gene regulation, complex interactions with other genetic and environmental factors, or even confounding variables, bias, or random chance in some studies [8]. Nevertheless, the significant findings in schizophrenia studies underscore the potential forensic psychiatric value of STRs [34].

Genetic data have been increasingly applied in forensic psychiatry algorithmic risk assessment models [35]. However, this research direction conflicts with the original premise that STR loci do not reveal medical privacy. If STR loci in forensic DNA databases can indeed reveal predispositions to conditions like schizophrenia, the privacy and security measures currently in place may no longer be adequate [36]. With forensic databases increasingly including individuals beyond traditional criminal populations, such as those involved in minor transgressions or even non-criminal groups, the need for updated legal frameworks to balance identification needs and health risk analysis has become critical [37].

Based on these considerations, this study aims to investigate whether commonly used forensic STR loci are associated with susceptibility to schizophrenia. If the results support such an association, STR loci may extend beyond their role in forensic identification, potentially implicating personal medical privacy. Our findings, along with existing research on the associations between STR loci and disease susceptibility, may prompt a re-evaluation of current privacy protection and data security standards within forensic DNA databases.

## 2. Materials and Methods

### 2.1. Sample Selection and Quality Control

The study was conducted in accordance with ethical guidelines and was approved by the Ethics Committee of the 904 Hospital of the People’s Liberation Army Joint Logistic Support Force. Participants included patients diagnosed with schizophrenia and control subjects, all recruited from the same hospital in Changzhou, China. The schizophrenia group consisted of 720 individuals (691 males and 29 females) selected from the Department of Psychiatry. All patients were diagnosed according to the Diagnostic and Statistical Manual of Mental Disorders, Fifth Edition (DSM-5) [38]. The control group, comprising 728 individuals (474 males and 254 females), had no history of mental disorders and was recruited from non-psychiatric departments of the hospital.

Prior to analysis, a quality control check was conducted to ensure data accuracy. Samples with incomplete or incorrect STR typing were excluded. As a result, 717 valid samples from the schizophrenia group (687 males and 30 females) and 725 valid samples from the control group (473 males and 252 females) were included in the final analysis.

### 2.2. DNA Extraction and Quantification

Genomic DNA was extracted from peripheral blood leukocytes using the QIAamp DNA Blood mini kit (QIAGEN GmbH, Hilden, Germany), according to the manufacturer’s instructions [39]. First, 20 µL of QIAGEN Protease and 200 µL of Buffer AL were added to 200 µL of blood sample, and the mixture was vortexed thoroughly and incubated at 56 °C for 10 min to lyse the cells. Following incubation, 200 µL of ethanol (96–100%) was added to the mixture, which was then vortexed to ensure homogeneity. The lysate was then loaded onto a QIAamp Mini spin column placed in a 2 mL collection tube and centrifuged at 8000 rpm for 1 min. The flow-through was discarded. The spin column was subsequently washed by 100% ethanol and two washing buffers (AW1 and AW2 buffers from the QIAamp DNA Blood Mini Kit). The genomic DNA was eluted by adding 200 µL of AE elution buffer to the column membrane by the simple diffusion method at room temperature for 1 min.

DNA quantification was performed using the Qubit™ 4 fluorometer with the Qubit™ dsDNA HS Assay kit (Thermo Fisher Scientific, Wilmington, DE, USA), following the manufacturer’s protocol [40,41]. Briefly, DNA concentrations were measured by preparing the standard samples and assay solution according to the Qubit™ protocol, and each sample was quantified to ensure it met the quality threshold for downstream PCR amplification.

### 2.3. PCR Amplification and Fragment Analysis

PCR amplification was carried out using the Goldeneye^®^ DNA Identification System 20A kit (Peoplespot, Beijing, China), following the manufacturer’s recommended protocol, for the multiplex amplification of 20 STR loci. The PCR reactions were prepared in a final volume of 10 μL, containing 4 μL of 2.5× PCR Master Mix, 2.0 μL of 5× Primer Mix, 0.16 μL of Taq Polymerase, and 1 ng of template DNA, with deionized water added to reach the 10 μL total volume. Amplification was conducted on a GeneAmp 9700 PCR System (Applied Biosystems, Foster City, CA, USA) under the following thermal cycling conditions: an initial denaturation at 96 °C for 2 min; 28 cycles of 94 °C for 5 s and annealing at 60 °C for 70 s; and a final extension at 60 °C for 30 min. The reactions were then held at 15 °C until further processing.

For fragment analysis, 1 μL of each amplified PCR product was mixed with 9 μL of a 19:1 solution of Hi-Di formamide (Thermo Fisher Scientific) and Goldeneye^®^ T500 size standard, consisting of fragments ranging from 65 to 500 nucleotides. The samples were denatured at 95 °C for 3 min and immediately cooled on ice at 0 °C for 3 min before loading onto the capillary electrophoresis system.

Capillary electrophoresis was conducted on a 3500XL Genetic Analyzer (Applied Biosystems), allowing for the precise separation of STR alleles based on size. GeneMapper ID-X Software v1.6 (Applied Biosystems) was used to analyze the fragment data, including allele calling, peak detection, and genotype assignment. Each genotyping result was manually reviewed to ensure accuracy and reliability across samples.

### 2.4. Gender Influence Analysis

Given that forensic STR typing includes the amelogenin locus for gender identification, we first analyzed gender differences to determine whether gender could influence associations at other loci. A chi-square test suggested that the approximation may not be accurate, due to the distribution of our data. The phi coefficient, calculated for further clarification, showed a relatively low value of 0.0203, indicating a weak correlation between gender and group classification. Despite Fisher’s exact test showing a highly significant result (*p*-value < 0.0001), the low phi coefficient suggests that this significance was likely due to the gender imbalance in the schizophrenia group, particularly the lower number of female participants. Consequently, the study focused on 19 core autosomal STR loci, including D3S1358, D6S1043, D13S317, Penta E, TH01, D18S51, D2S1338, CSF1PO, Penta D, D16S539, vWA, D21S11, D7S820, D5S818, TPOX, D8S1179, D12S391, D19S433, and FGA.

### 2.5. Statistical Analysis

The Hardy–Weinberg equilibrium (HWE) for both the schizophrenia and control populations was tested using the HardyWeinberg package in R software (version 4.4.1) [42]. The chi-square test, phi coefficient, and Fisher–Freeman–Halton exact test (Fisher’s exact test) were conducted using R software. These analyses were employed to assess the relationship between gender distribution and schizophrenia in the sample. Due to the low allele/genotype counts (<5) in both groups, Fisher’s exact test with Monte Carlo significance (95%, two-sided) was employed instead of the chi-square test for all comparisons. A Bonferroni correction was applied to adjust for multiple comparisons, setting the significance threshold at 0.05/19 = 0.0026. Data visualization was performed using GraphPad Prism (version 10, GraphPad Software, San Diego, CA, USA).

## 3. Results

### 3.1. Hardy–Weinberg Equilibrium and Comparison of 19 STR Loci

No significant deviations from the Hardy–Weinberg equilibrium (*p* > 0.05) were observed in either the schizophrenia or control group. The results of the equilibrium tests, along with Fisher’s exact tests for allele and genotype frequencies across the 19 STR loci, are presented in Table 1.

### 3.2. Allele Frequency Distributions of D3S1358, D13S317, and TPOX Loci

**Figure 1:**
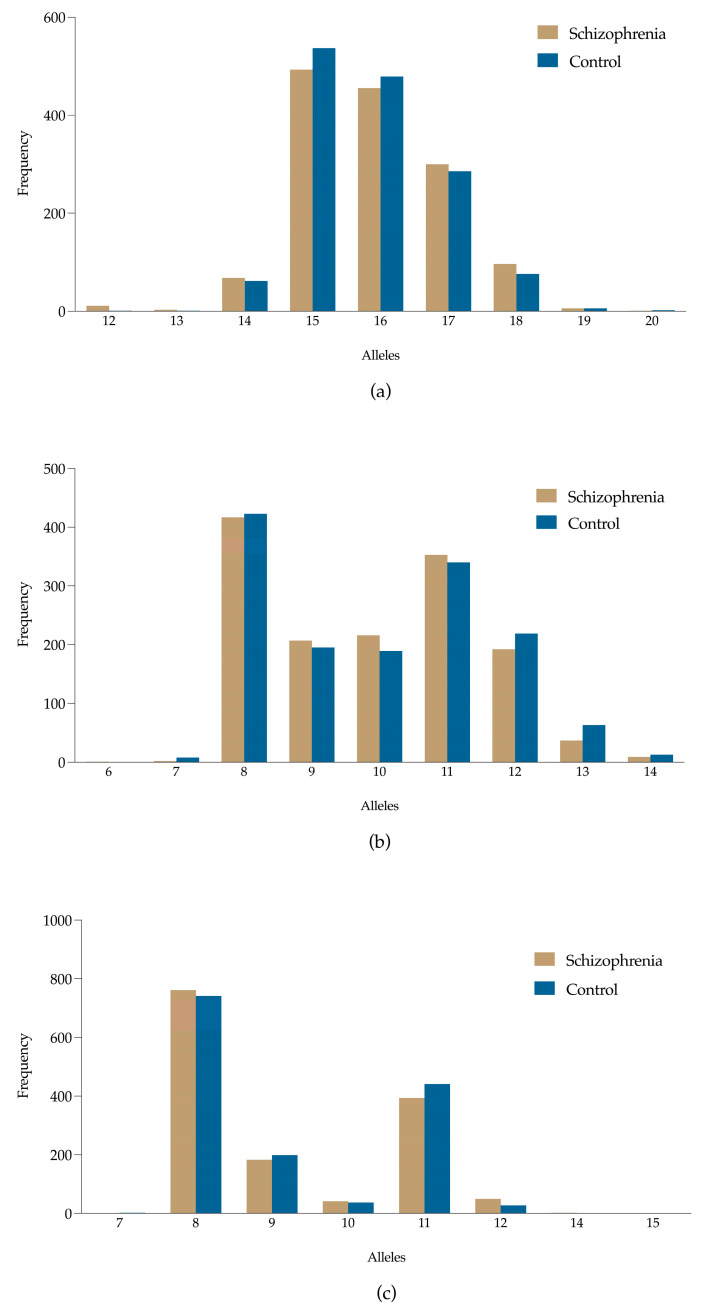
Frequency distributions of alleles for D3S1358, D13S317, and TPOX in control and schizophrenia groups. (**a**) Allele frequencies of D3S1358; (**b**) allele distribution of D13S317; (**c**) allele frequencies of TPOX.

The allele frequency distributions for the D3S1358, D13S317, and TPOX loci are presented in Table 2. Initial analyses revealed some differences between the schizophrenia and control groups for these loci. At the D3S1358 locus, allele 15 was the most frequent in both the schizophrenia (34.38%) and control (37.03%) groups. The frequencies of alleles 16 and 17 were also quite similar between the two groups, with no substantial variation observed in their distribution. For the D13S317 and TPOX loci, the allele distributions were nearly identical between the schizophrenia and control groups, showing no significant differences in frequency (see Figure 1). The allele distributions at these loci further confirm the lack of notable variation between the two populations.

Despite these initial observations, none of the differences were substantial enough to suggest a strong association between these loci and schizophrenia. To account for multiple comparisons across the 19 loci, a Bonferroni correction was applied to the *p*-values. After applying the correction, none of the observed differences in allele frequencies at the D3S1358, D13S317, or TPOX locus remained statistically significant (corrected *p* < 0.0026 for all loci).

These findings indicate that while minor differences in allele frequencies were observed between the schizophrenia and control groups, these variations are likely due to random fluctuations. Further investigation with larger samples and more diverse populations may be required to explore any subtle genetic influences at these loci.

## 4. Discussion

The potential for forensic STR loci to provide medically relevant phenotypic information has garnered increasing attention, particularly regarding their association with psychiatric conditions like schizophrenia. While forensic STRs were traditionally selected to avoid medical relevance, recent studies suggest that some loci, especially those located within introns or near regulatory genes, may influence gene expression and impact disease susceptibility. In this study, we examined 19 STR loci commonly used in forensic identification, comparing 717 schizophrenia patients to 725 controls, both drawn from a large hospital with diverse demographic representation. This sample selection aimed to reflect the general characteristics of schizophrenia patients and the local population in this region, enhancing the representativeness and relevance of our findings for broader forensic and genetic studies. No significant deviations from the Hardy–Weinberg equilibrium were observed, and initial differences observed at some loci were not sustained after correction for multiple comparisons.

Schizophrenia is known to be highly polygenic, with numerous genetic factors contributing to its development, and these effects may vary across populations [34]. In our study, despite previous reports suggesting the involvement of up to 14 STR loci (see the Section 1) in schizophrenia, we did not observe any consistent patterns. This could be attributed to genetic heterogeneity across different populations or variations in study designs, as well as the high influence of environmental factors in modulating genetic variants’ effects. Our results align with the current understanding that STRs used in forensic applications were not designed for medical diagnostics, and their potential utility in identifying psychiatric conditions remains speculative. These complexities highlight the need for further validation of our results through larger, more diverse cohort studies.

Despite our study finding no significant association between the TH01 locus and schizophrenia, it is worth noting that TH01 has been one of the most frequently studied loci in relation to this disorder, with findings often varying across populations. For instance, a Japanese study [11] reported lower frequencies of the TH01-9 and TH01-6 alleles in female schizophrenia patients, while studies by Jacewicz et al. [9,10,43] in Poland found a higher frequency of the TH01-7 allele and a lower frequency of the TH01-9.3 allele in schizophrenia patients, compared to controls. These findings suggest that the association between TH01 polymorphisms and schizophrenia may be influenced by population-specific genetic and environmental factors, rather than being universally applicable. This variability highlights the importance of conducting research across diverse populations to fully understand the role of STRs like TH01 in schizophrenia.

TH01 remains an important candidate in neuropsychiatric research due to its relationship with the *TH* gene. The *TH* gene, which is involved in dopamine regulation, has been extensively studied in relation to neuropsychiatric diseases, including schizophrenia, bipolar disorder, depressive disorder, and anxiety disorder [44]. In vitro studies have shown that TH01 may exert a quantitative silencing effect by regulating the transcription of the *TH* gene, with the extent of this inhibitory effect being positively correlated with the number of repeats in the TH01 allele [24]. In other words, longer TH01 repeat sequences (e.g., allele 10) may more significantly suppress *TH* gene expression, potentially leading to reduced dopamine synthesis. Therefore, longer TH01 alleles may be more prevalent in individuals with dopamine-related dysfunctions, such as those exhibiting aggressive behavior [32], while shorter alleles might be associated with normal dopamine levels and thus confer a protective effect.

While TH01 has been widely studied in relation to schizophrenia, evidence linking other STR loci to this disorder has been more limited. For example, D16S539 has been suggested as a potential candidate, due to its location on chromosome 16q24.1, a region implicated in neurodevelopmental disorders and associated with intellectual disabilities and developmental delays [45]. The potential involvement of nearby regulatory elements or non-coding RNAs, such as LINC00917, in brain development and mental health disorders has not been definitively confirmed, leaving the possible mechanisms linking D16S539 to schizophrenia still unclear and requiring further investigation.

The implications of our findings extend beyond schizophrenia research. Since the 1990s, forensic DNA databases have played a crucial role in criminal investigations, using STR loci to identify individuals. However, the emerging evidence linking certain STRs to disease susceptibility raises important privacy concerns, as forensic data may inadvertently reveal sensitive health information. This necessitates a reassessment of the guidelines governing the use of forensic DNA databases to ensure that they protect against the misuse of medical information, which could lead to discrimination or other negative consequences. Our findings provide valuable reference points for forensic genetic markers more broadly, especially as forensic applications increasingly incorporate Y-STRs, mitochondrial DNA, and a growing array of SNPs.

Finally, while this study did not find consistent associations between STR profiles and schizophrenia, it is important to note that these findings are based on association analysis and do not completely rule out the possibility of a relationship. The proposed mechanisms, particularly those involving the *TH* gene and dopamine regulation, still warrant further investigation. Based on our data and previous publications, it seems unlikely that STR profiling alone, as it currently stands, could reliably identify individuals with schizophrenia. Although certain studies have suggested associations in specific populations, these findings lack consistency across diverse groups. Therefore, while forensic STR loci may hold potential, current evidence suggests that their utility in determining psychiatric conditions such as schizophrenia remains speculative and would require further validation. As forensic science adopts more advanced sequencing technologies, future research with larger and more diverse samples is needed to clarify the relationships between STR loci and psychiatric disorders and to explore their broader implications for forensic and medical genomics.

## 5. Conclusions

This study did not find significant associations between the analyzed forensic STR loci and schizophrenia. However, the lack of significant findings in our study does not entirely rule out the possibility of associations between these loci and psychiatric conditions. Previous research, particularly on the TH01 locus, continues to suggest its relevance in neuropsychiatric disorders, due to its role in dopamine regulation. The complexity of genetic contributions to schizophrenia, combined with population-specific variability, reinforces the need for further investigation with larger and more diverse cohorts. Beyond genetic associations, our findings raise important ethical and privacy considerations. Forensic DNA databases, initially developed for criminal identification, are increasingly used in broader contexts, with emerging evidence suggesting that certain STR loci may reveal medically relevant information. This potential dual use of forensic DNA data underscores the need to reevaluate current legal and ethical frameworks to ensure the protection of individual privacy rights while balancing public safety needs. Future research should persist in exploring these genetic associations, and policymakers should navigate the delicate equilibrium between public safety and individual privacy rights. Overall, our study emphasizes the evolving role of STR loci in forensic genetics and the necessity for guidelines that adapt to these emerging realities.

## Figures and Tables

**Table 1:** HWE and Fisher’s exact test *p*-values for allele and genotype comparisons between schizophrenia and control groups across 19 STR loci.

STR	Schizophrenia HWE-*p*	Control HWE-*p*	Allele Fisher-*p* ^1^	Genotype Fisher-*p* ^2^
D3S1358	0.6673	0.6623	0.0365	0.4579
D6S1043	0.2895	0.4032	0.9639	0.5520
D13S317	0.2897	0.5134	0.0332	0.0825
Penta E	0.8561	0.1464	0.5316	0.0381
TH01	0.1719	0.2792	0.4401	0.7627
D18S51	0.7118	0.2055	0.3929	0.1826
D2S1338	0.4025	0.1177	0.8525	0.9520
CSF1PO	0.8570	0.7528	0.3880	0.7514
Penta D	0.2666	0.4112	0.9979	0.9789
D16S539	0.7509	0.2885	0.4223	0.2831
vWA	0.4424	0.9972	0.4633	0.7761
D21S11	0.4602	0.5158	0.7409	0.5459
D7S820	0.6633	0.9355	0.5852	0.9348
D5S818	0.1876	0.5141	0.7231	0.4079
TPOX	0.9957	0.9282	0.0237	0.3918
D8S1179	0.6169	0.1156	0.2312	0.0636
D12S391	0.2351	0.4518	0.2533	0.1182
D19S433	0.8145	0.3474	0.2986	0.3373
FGA	0.1229	0.7229	0.7133	0.3778

^1,2^ Fisher’s exact test p-values reflect comparisons between the schizophrenia and control groups.

**Table 2:** Allelic counts and frequencies (%) of D3S1358, D13S317, and TPOX loci in schizophrenia and control groups.

Allele	D3S1358		D13S317		TPOX	
	Schizophrenia	Control	Schizophrenia	Control	Schizophrenia	Control
	N = 717 (%)	N = 725 (%)	N = 717 (%)	N = 725 (%)	N = 717 (%)	N = 725 (%)
6			1 (0.07%)	#N/A		
7			2 (0.14%)	8 (0.55%)		2 (0.14%)
8			417 (29.08%)	423 (29.17%)	762 (53.14%)	741 (51.10%)
9			207 (14.44%)	195 (13.45%)	183 (12.76%)	199 (13.72%)
10			216 (15.06%)	189 (13.03%)	42 (2.93%)	38 (2.62%)
11			353 (24.62%)	340 (23.45%)	394 (27.48%)	442 (30.48%)
12	11 (0.77%)	1 (0.07%)	192 (13.39%)	219 (15.10%)	50 (3.49%)	28 (1.93%)
13	3 (0.21%)	1 (0.07%)	37 (2.58%)	63 (4.34%)		
14	68 (4.74%)	62 (4.28%)	9 (0.63%)	13 (0.90%)	2 (0.14%)	
15	493 (34.38%)	537 (37.03%)			1 (0.07%)	
16	455 (31.73%)	479 (33.03%)				
17	300 (20.92%)	286 (19.72%)				
18	97 (6.76%)	76 (5.24%)				
19	6 (0.42%)	6 (0.41%)				
20	1 (0.07%)	2 (0.14%)				

## Data Availability

The data presented in this study are available upon request from the corresponding author due to privacy and ethical restrictions.
